# A novel transplantable model of lung cancer-associated tissue loss and disrupted muscle regeneration

**DOI:** 10.1186/s13395-020-00225-6

**Published:** 2020-03-09

**Authors:** Paige C. Arneson-Wissink, Alexandra M. Ducharme, Jason D. Doles

**Affiliations:** Department of Biochemistry and Molecular Biology, Mayo Clinic, Rochester, MN 55905 USA

**Keywords:** Cancer-associated muscle wasting, Cachexia, Lung cancer, Skeletal muscle

## Abstract

**Background:**

Cancer-associated muscle wasting (CAW), a symptom of cancer cachexia, is associated with approximately 20% of lung cancer deaths and remains poorly characterized on a mechanistic level. Current animal models for lung cancer-associated cachexia are limited in that they (1) primarily employ flank transplantation methods, (2) have short survival times not reflective of the patient condition, and (3) are typically performed in young mice not representative of mean patient age. This study investigates a new model for lung cancer-associated cachexia that can address these issues and also implicates muscle regeneration as a contributor to CAW.

**Methods:**

We used tail vein injection as a method to introduce tumor cells that seed primarily in the lungs of mice. Body composition of tumor-bearing mice was longitudinally tracked using NMR-based, echo magnetic resonance imaging (echoMRI). These data were combined with histological and molecular assessments of skeletal muscle to provide a complete analysis of muscle wasting.

**Results:**

In this new lung CAW model, we observed (1) progressive loss in whole body weight, (2) progressive loss of lean and fat mass, (3) a circulating cytokine/inflammatory profile similar to that seen in other models of CAW, (4) histological changes associated with muscle wasting, and (5) molecular changes in muscle that implicate suppression of muscle repair/regeneration. Finally, we show that survival can be extended without lessening CAW by titrating injected cell number.

**Conclusions:**

Overall, this study describes a new model of CAW that could be useful for further studies of lung cancer-associated wasting and accompanying changes in the regenerative capacity of muscle. Additionally, this model addresses many recent concerns with existing models such as immunocompetence, tumor location, and survival time.

## Background

Cancer cachexia is a complex syndrome associated with approximately 20% of lung cancer deaths; a hallmark symptom of cancer cachexia is cancer-associated muscle wasting (CAW) [1, 2]. Although this syndrome is associated with negative patient outcomes, it remains poorly understood on a mechanistic level. Many groups have investigated the inflammatory environment associated with CAW, in particular, inflammatory cytokines such as tumor necrosis factor alpha (TNFα) and interleukin 6 (IL6). Despite considerable time invested in this area, clinical trials targeting the inflammatory microenvironment alone have been unsuccessful [3, 4]. Other groups have focused on the proteolytic environment in muscle as a potential driver of CAW, implicating both autophagy and ubiquitin-mediated pathways [5–7]. The current mechanistic understanding of CAW is largely based off of work done in mouse models, most notably the Lewis lung carcinoma (LLC) and C-26 models.

The LLC model of CAW relies on the transplantation of LLC1 non-small cell lung cancer cells, derived from C57/B6 mice, into syngeneic, and thus immunocompetent, C57/B6 recipient mice. The LLC model is one of the few syngeneic and reproducible models for lung cancer/lung cancer-associated wasting available today [8]. Most commonly, LLC1 cells are implanted via flank injection, which results in large primary tumors around 7 days post-implantation. A second popular model for CAW, the C26 model, was first described in 1990 and utilizes flank injection of colon 26 carcinoma cells [9]. Like the LLC model, this model displays elevated expression of proteasome components, which contribute to loss of muscle mass [10]. The C26 model presents with longer median survival (25 days) than the LLC model [10]. Notably, both the C26 and LLC models share a common weakness in that tumors do not arise in the tumor tissue of origin. Although recent studies have attempted to correct this issue using autochthonous genetically engineered mouse models (GEMMS), their utility and flexibility are limited. For example, in pancreatic cancer, there have been multiple efforts to create CAW models that focus on tumors arising in their native tissue via either genetic manipulation or orthotopic implantation [11–15]. The benefits and limitations of these models are addressed in more depth in the discussion. Despite these advances in the context of pancreas cancer, lung cancer models have lagged behind. Orthotopic transplantation has been established in rodent models, but requires an invasive surgery [16, 17]. Furthermore, many genetic methods to derive orthotopic tumors in lungs require viral delivery of Cre recombinase [18]. Complications arising in both these models are high and prompted us to develop a simple, reproducible, and scalable model of lung CAW.

We identified the need for better models of CAW that met the following criteria: (1) immunocompetent environment, (2) tumors arising in the appropriate/matching tissue of origin, and (3) ability to modulate the timing/duration of wasting. Here, we present studies on a novel mouse model for lung CAW, which has met these criteria. This model utilizes syngeneic, immunocompetent mice and tail-vein injection of cells as a modality for lung tumor seeding; shows a median survival of approximately 26 days that can be modulated as a function of injected cell number; and exhibits hallmarks of lung CAW. The model presented herein fills a gap in models for studying CAW and will be a valuable resource for the wasting community moving forward.

## Methods

### Animals

Mice were bred and housed according to the NIH guidelines for the ethical treatment of animals in a pathogen-free facility at the Mayo Clinic (Rochester, MN campus). Mayo Clinic’s Institutional Animal Care and Use Committee (IACUC) approved all animal protocols. For 393P-induced CAW model, 150 μL 393P cells (containing 3 × 10^6^, 1 × 10^6^, or 3 × 10^5^ cells depending on group), or an equal volume of PBS (Gibco, 14190-144) (control), was injected via tail vein into 7-week-old male 129S2/SvPasCrl mice (Jackson Laboratories, Bar Harbor, ME). Development of cachexia was monitored twice weekly by body weight, fat, and lean mass. Mice were euthanized when endpoint criteria (weight loss greater than or equal to 20% of body weight, inability to ambulate, inability to reach food and/or water, tumors greater than or equal to 10% of body weight, tumors that have ulcerated, a body condition score of 1 or less using the IACUC approved scoring system) were reached; therefore, animals were euthanized on a case-by-case basis. For some animals, their decline in health was rapid and resulted in spontaneous death. Survival time was reported as the time to reach endpoint criteria, or time until spontaneous death. Note that the criteria “tumors greater than or equal to 10% of body weight, tumors that have ulcerated” do not apply to primary lung tumors, but were used as assessment for palpable metastases that formed on the back and hind limbs of some animals. Blood serum, heart muscle, lungs, tibialis anterior (TA), gastrocnemius (GR), and epididymal fat pad were collected immediately for analyses. Exact *n* for each experiment is included in the figure legends.

### Animal imaging

Body weight, lean mass, and fat mass were measured twice weekly until the endpoint. A baseline measurement was taken prior to the start of the study. Body composition was measured using an NMR-based echo magnetic resonance imaging EchoMRI-100/130 system (EchoMRI, Houston, TX, USA).

### Grip strength testing

Bioseb InVivo Research Instruments grip strength meter, model GT3, was used to measure grip strength of mice at their survival endpoint. Vehicle mice were assessed at the same time. We utilized the bar attachment for the grip strength meter. Each mouse had 3 total front limb grips, with resting time between each run. The 3 grips were averaged into a single data point for each mouse. Grips were not included in the final count of 3 grips if the mouse used their hind limbs, gripped with only one paw, or refused to grip.

### Cell culture

Kras^LA1/+^;p53^R172H/Δg/+^ lung adenocarcinoma cell line (393P) was generated as previously described [19]. 393P cells were grown on tissue culture-treated dishes in growth media consisting of DMEM media (Gibco #11995-065, lot 2051518) supplemented with 10% fetal bovine serum (Gibco #10099131, lot 2017488) and antibiotics. Cells were validated by IDEXX analytics and confirmed to be a pure culture and murine in origin. Cells were removed from the plate with TrypLE (Gibco, 12604-013), counted, and resuspended in PBS prior to injection.

### Immunostaining

Left TA whole muscle was prepared for O.C.T embedding and cryosectioned. Tissue sections (8–10 μm) were post-fixed in 4% paraformaldehyde for 5 min at room temperature prior to immunostaining. Once fixed, isolated tissue sections were permeabilized with 0.5% Triton X-100 in PBS, followed by blocking with 3% BSA in PBS. Primary antibody incubations occurred at RT for 90 min or overnight at 4°, and secondary antibody incubations followed at RT for 45 min in 3% BSA in PBS. The following antibodies were used in this study: laminin (Sigma 4HB-2) and Pax7 (developmental hybridoma bank). Secondary antibodies were all Alexa fluorescent conjugates (488, 555, or 647) from Invitrogen or Jackson ImmunoResearch. Stained tissue sections were imaged on a Nikon Eclipse Ti-U camera and microscope system. Acquired images were analyzed for myofiber feret diameters using MyoVision and manual colocalization quantification using ImageJ.

### Cytokine studies

Blood serum was collected from mice at the endpoint. The MD31 cytokine panel was performed by Eve Technologies (Eve Technologies, Calgary, AB Canada), and samples were prepared as recommended by Eve Technologies. All samples were run in technical duplicate.

### Quantitative RT-PCR

Total gastrocnemius RNA was isolated, purified, and DNase I treated using TRIzol and purified on RNeasy Mini kit columns (Qiagen, Mississauga, ON, Canada). RNA was quantified using a NanoDrop Spectrophotometer (Thermo Scientific, Wilmington, DE, USA). Two micrograms of total RNA was reverse transcribed with primers using the High-Capacity cDNA Reverse Transcription Kit (Life Technologies, Carlsbad, CA, USA). QPCR was performed on a ViiA7 Quantitative PCR System (Applied Biosystems by Life Technologies, Austin, TX, USA). All samples were run in technical triplicate. Standard delta delta CT analysis was used post-PCR. Primer sequences are available upon request.

### Lung and heart histopathology

At endpoint, lung and heart tissues were fixed in 4% paraformaldehyde. After 24 h, tissue was moved to 70% ethanol. Lung tissue was embedded in paraffin, and tissue sections (6 μm) were incubated at 37° for 60 min prior to staining with hematoxylin and eosin (H&E). Stained lung tissue sections were analyzed by GEMpath Inc. (Dr. Brad Bolon, Longmont, CO) and imaged on a Nikon Eclipse Ts2 microscope. Samples could be segregated with 100% reliability at using both the naked eye and the microscope. Heart tissue was embedded in paraffin, and tissue sections (6 μm) were incubated at 37° for 60 min prior to post-fixation in Bouin’s solution, followed by staining with Picro Sirius Red. Stained tissue sections were imaged on a Motic EasyScan slide scanner and analyzed using ImageJ thresholding in the green channel.

### RNA sequencing

RNA sequencing was done in collaboration with the Mayo Clinic Medical Genome Facility Genome Analysis Core and Mayo Clinic Bioinformatics Core. Transcripts with RPKM intensities equal to zero in all samples were removed, and the remaining transcripts’ RPKM values were log2 transformed and used for subsequent analyses. Hierarchical clustering was performed using TIBCO Spotfire software. Clusters were formed by complete linkage clustering, and distances were measured by correlation. Principal component analysis (PCA) was performed using TIBCO Spotfire. RNAseq data set has been deposited in the Sequence Read Archive, as described in the “Data availability” section.

### Statistics

Data are represented as the mean ± SD using GraphPad Prism (GraphPad Software, San Diego, CA) unless noted otherwise in the figure legends. Quantification of muscle cross-sections using minimum feret diameter measurements was analyzed by non-linear regression (least-squares method) and compared between conditions using an extra-sum-of-squares *F* test. All other comparisons between groups were performed using unpaired two-tailed Student’s *t* tests or mixed-effects analysis, as noted in figure legends. For all analyses, a *p* < 0.05 was considered significant (denoted with asterisk).

### Data availability

The RNAseq dataset generated and analyzed during the current study is available in the Sequence Read Archive (SRA) (National Center for Biotechnology Information, NCBI) via the following link:  https://www.ncbi.nlm.nih.gov/sra/PRJNA604626, BioProject ID PRJNA604626. All other datasets generated during the current study are available from the corresponding author upon request.

## Results

### Development of a transplantable model for lung cancer-associated tissue loss

**Fig. 1:**
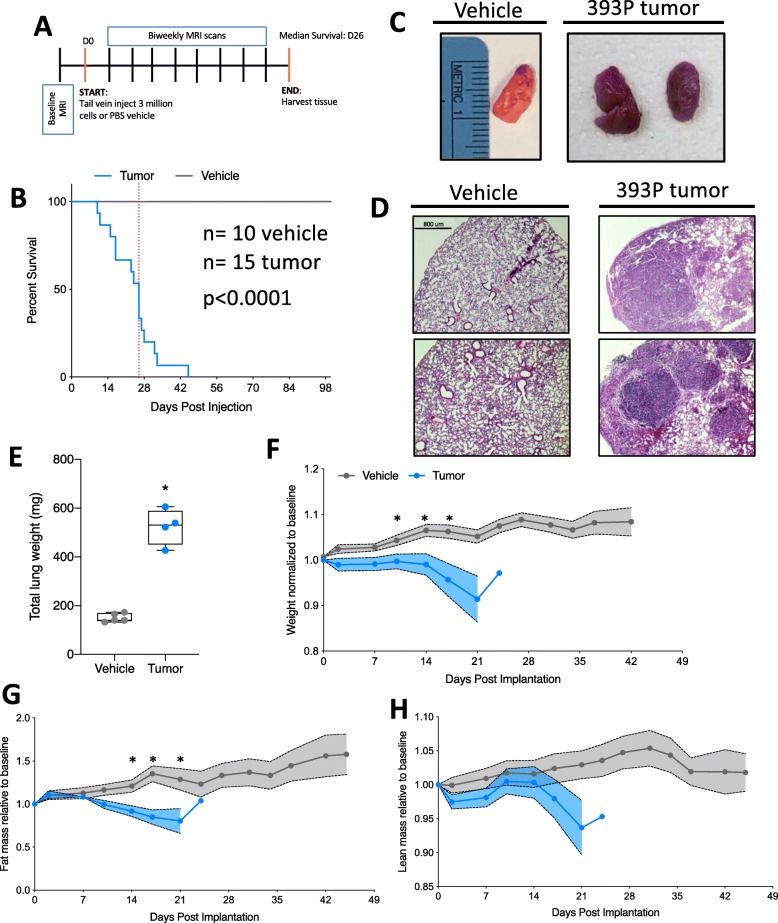
Transplantable model for lung cancer-associated tissue loss. **a** Schematic of the study design. D0 = day tumor cells were injected into mice. All studies were survival studies; therefore, endpoint was variable. The median survival was 26 days. echoMRI scans were completed twice per week during the course of the study. **b** Survival curve for vehicle and tumor injected cells. Dotted vertical line indicates the median survival (26 days). *n* = 10 vehicle, 15 tumor. Data were compiled from 3 independent cohorts of animals. Survival is statistically different (*p* < 0.0001) by Gehan-Breslow-Wilcoxon test. **c** Representative images of lungs from vehicle and tumor-bearing mice. Ruler reference in centimeters. **d** Representative hematoxylin and eosin (H&E) staining of lung tissue from vehicle (left) and tumor-bearing (right) mice. Two representative images from 2 individual animals in each condition. Scale bar is 800 μm. **e** Total lung weight (all lobes) from vehicle and tumor-bearing mice. Individual points represent individual animals. Boxes represent the inner quartiles, and whiskers represent the minimum and maximum values. **f** Total mouse weight across the study, normalized to pre-tumor baseline weight. Error bars are SEM. *p* values presented in the figure are the result of mixed-effects analysis, with Geisser-Greenhouse correction. Significance at individual points was determined by correction for multiple testing using false discovery rate (Benjamini and Hochberg). **g** echoMRI calculated total fat mass across study, normalized to pre-tumor baseline fat mass. Error bars are SEM, and statistical analysis is as described above. **h** echoMRI calculated total lean mass across study, normalized to pre-tumor baseline lean mass. Error bars are SEM, and statistical analysis is as described above. *p < 0.05 by Student’s *t* test *n* = 5 vehicle, 4 tumor-bearing 7-week-old male 129S2/SvPasCrl mice for all data except survival curve

Given the increasing need in the CAW field for models featuring tumors arising in their natural location, we sought to develop such a model in the context of lung cancer. To accomplish this, we used tail vein injection as a method whereby injected lung tumor cells implant primarily in the lungs [20]. We selected a cell line that was derived from Kras^LA1/+^;p53^R172H/Δg/+^ mice bearing lung adenocarcinomas [19]. Cells were cultured more than 10 passages prior to injection. For the injection, cells were suspended in PBS and control animals were injected with PBS vehicle only. Throughout the course of the study, we assessed body composition via echoMRI until humane endpoint (see experimental schematic in Fig. 1a). We performed survival studies on 3 cohorts of mice (total *n* = 10 vehicle and 15 tumor) and observed significantly decreased survival for tumor-bearing mice (average survival tumor = 26.2 days) (Fig. 1b). Upon macroscopic inspection of the lungs, tumor-bearing mice had many small nodules, fibrotic tissue, and increased tissue density (Fig. 1c). Upon microscopic inspection of the lungs in tumor-bearing mice, alveolar fields were replaced by multiple, coalescing tumor nodules (> 70% in 3 animals, 40–50% in 2 animals assessed). The tumor nodules consisted of loosely packed, vacuolated cells and in some cases were associated with small clusters of intra-tumoral lymphocytes, usually at the margins of blood vessels or nodules. Lastly, the pleural surface was roughened by projecting fronds/plaques of tumor cells. In comparison, vehicle animals displayed expanded alveolar spaces and a smooth pleural surface (Fig. 1d). Furthermore, we observed a statistically significant increase in total lung weight in tumor-bearing mice (Fig. 1e). Due to the proximity of the lungs and heart, and an established literature base regarding cardiac cachexia, we assessed cardiac fibrosis [21]. Cardiac tissue stained with Picro Sirius Red did not show any difference in fibrosis between tumor-bearing and vehicle animals (Supplemental Figure 1 A-C).

Concurrent with evaluations of tumor effects on overall survival, we performed longitudinal body composition analyses. First, we observed a significant difference in time and treatment co-variance for body weight, which was highlighted by significant decreases in body weight at 10, 14, and 17 days post-tumor implantation (Fig. 1f). Next, we observed a significant difference in time and treatment co-variance for total fat mass, which was highlighted by significant decreases in fat mass at 14, 17, and 21 days post-tumor implantation (Fig. 1g). Lastly, we observed a significant difference in time and group co-variance in total lean mass, although individual days had no significant difference (Fig. 1h). Overall, there were decreases in total body weight, total fat mass, and total lean mass as the tumor progressed (the latter two variables measured using echoMRI) (Fig. 1f–h).

### Lung cancer-associated molecular changes in serum and skeletal muscle

**Fig. 2:**
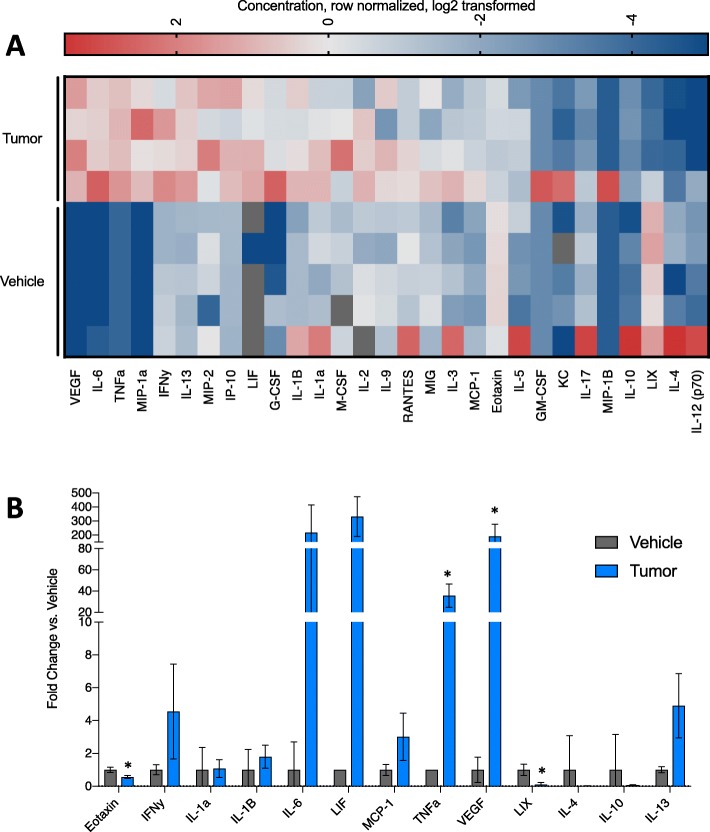
Lung cancer-associated inflammation. **a** Heat map of 29 cytokines profiled in the serum of vehicle or tumor-bearing mice. Features are sorted by the highest average of the tumor samples, from top to bottom. Values are the concentration, row normalized for each feature, then log2 transformed. Red = high expression, blue = low expression. **b** Bar graph of cytokines commonly associated with muscle wasting and/or cancer cachexia. Individual points represent individual animals. Boxes represent the inner quartile, and whiskers represent the minimum and maximum values. **c** Bar graph of cytokines significantly upregulated in tumor-bearing animals. Individual points represent individual animals. Boxes represent the inner quartile and whiskers represent the minimum and maximum values. *p < 0.05 in multiple *t* test with Holm-Sidak multiple testing correction. *n* = 5 vehicle, 4 tumor-bearing 7-week-old male 129S2/SvPasCrl mice

Loss in both body weight and lean mass is a hallmark of CAW. Many studies have implicated heightened inflammatory signaling as a driver of CAW [22–24]. We quantitatively assessed concentrations of 31 cytokines in the serum of tumor-bearing and vehicle animals (EveTechnologies) (*n* = 5 vehicle and 4 tumor). Ranked analysis (vertically by their average in the tumor-bearing animals; highest to lowest) revealed substantial differences between experimental groups (Fig. 2a). Specifically, we observed significant differences in 4 factors: eotaxin (down ~ 2-fold), C-X-C motif chemokine 5 (LIX, down ~ 10-fold), TNFα (up ~ 30-fold), and vascular endothelial growth factor (VEGF, up ~ 190 fold) (Fig. 2c). Of note, TNFα accumulation has been previously implicated in CAW [22, 25].

**Fig. 3:**
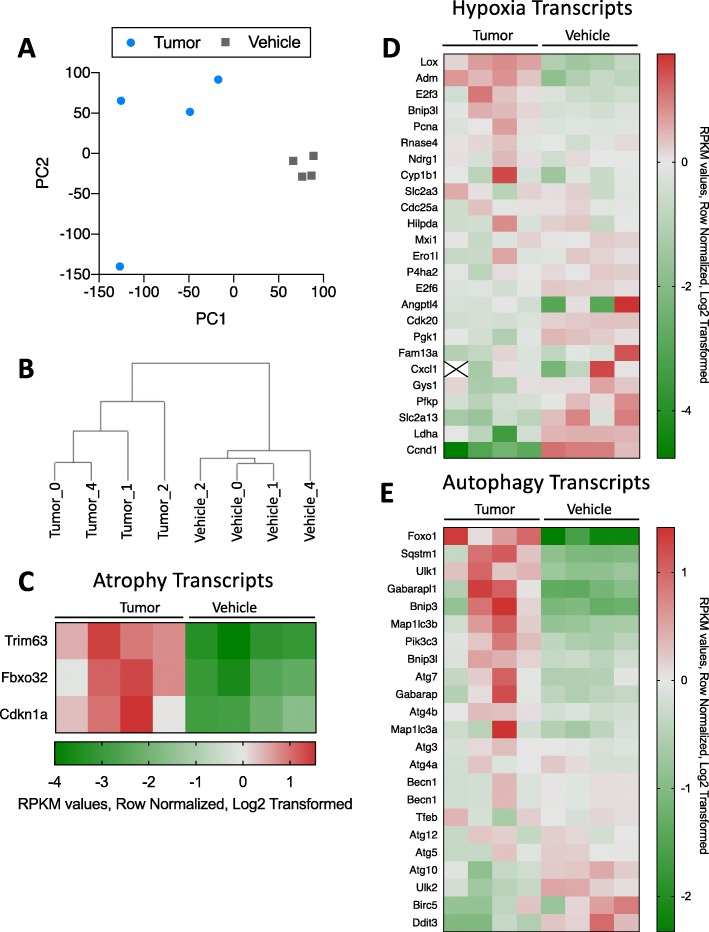
Lung cancer-associated transcriptional changes. **a** PCA analysis of all transcripts detected in tumor-bearing and vehicle mouse muscle. **b** Hierarchical clustering separates tumor-bearing and vehicle mouse muscle based on differential transcriptome patterns. **c** Heat map of selected transcripts commonly associated with muscle atrophy. Trim63 was significantly upregulated in tumor-bearing mouse muscle *p* = 0.011. **d** Heat map of selected transcripts commonly associated with the hypoxia pathway. Of particular interest, 5 genes were significantly downregulated (Cdk20 *p* = 0.0004, Ccnd1 *p* = 0.0024, E2F6 *p* = 0.0392) or upregulated (Adm *p* = 0.0081, Lox *p* = 0.0095). **e** Heat map of select4ed transcripts commonly associated with the autophagy pathway. There were no significant differences in the expression of these genes. For all heat maps, features are sorted by the highest average of the tumor samples, from top to bottom. Values are the RPKM values, row normalized for each feature, and then log2 transformed. Red = high expression, green = low expression. Statistical significance was determined as *p* < 0.05 in multiple *t* test with Holm-Sidak multiple testing correction. *n* = 4 vehicle, 4 tumor

In addition to serum cytokine profiling, we were interested in the transcriptional changes occurring in skeletal muscle, likely in response to these inflammatory changes. We therefore assessed the transcriptome of whole muscle from tumor-bearing and vehicle mice using RNA sequencing (RNAseq). Principal component analysis (PCA) and hierarchical clustering separated tumor-bearing from vehicle mice (Fig. 3a, b, Supplemental Figure 2). We then assessed differential expression of selected transcripts relevant to CAW-associated pathways including muscle atrophy, autophagy, and hypoxia (Fig. 3c–e). We observed a significant (*p* < 0.05, multiple *t* test with Holm-Sidak multiple testing correction) ~ 20-fold increase in Trim63 (a transcript encoding MuRF1, an E3 ubiquitin ligase associated with muscle atrophy), in tumor-bearing mouse muscle (Fig. 3c). Expression of Fbxo32 and Cdkn1a was also elevated, although these differences did not achieve statistical significance (*p* = 0.058 and *p* = 0.1717, respectively) in our analysis. Among selected hypoxia-related transcripts, we observed two significantly upregulated transcripts (Lox and Adm), and three significantly downregulated transcripts (Cdk20, E2f6, and Ccnd1) in tumor-bearing versus vehicle mice (Fig. 3d). Finally, while many autophagy transcripts were elevated in most tumor-bearing mice, we did not observe significant pair-wise differences in any of the selected autophagy-related transcripts shown (Fig. 3e).

### Lung cancer-associated muscle loss

**Fig. 4:**
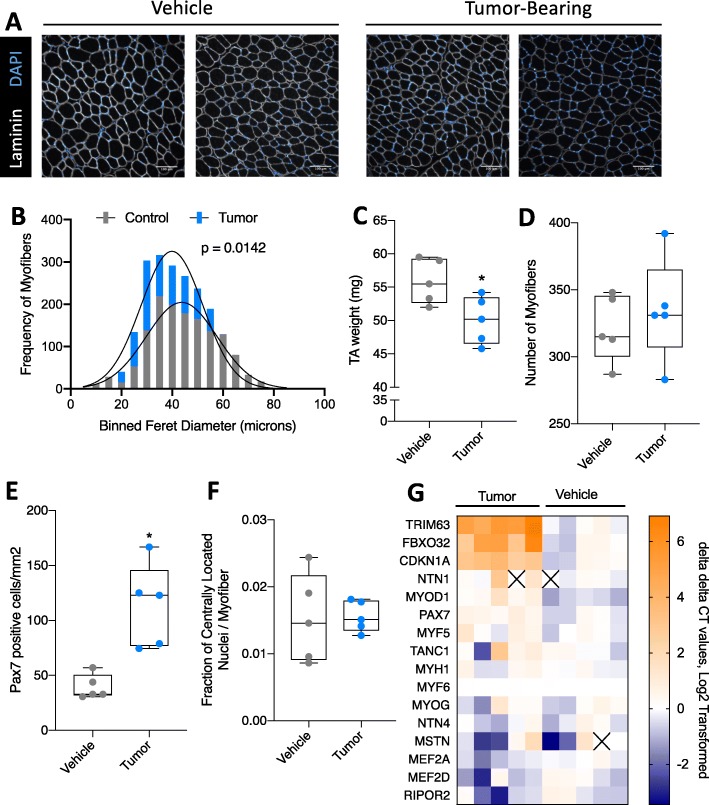
Lung cancer-associated muscle loss. **a** Representative images of vehicle (left two images) and tumor-bearing (right two images) tibialis anterior (TA) muscle cross-sections. Immunofluorescent staining for laminin (myofiber membranes, white) and DAPI (nuclei, blue). **b** Quantification of minimum feret diameters of myofibers from tumor-bearing and vehicle mice. Feret diameters were binned to a histogram and fit with a non-linear regression (Gaussian, least squares regression). Myofibers of tumor-bearing animals were significantly smaller than vehicle; *p* = 0.0142. **c** Wet weight of TA muscle from vehicle and tumor-bearing animals. Individual points represent individual animals. Boxes represent the inner quartile, and whiskers represent the minimum and maximum values. **d** Wet weight of gastrocnemius (GR) muscle from vehicle and tumor-bearing animals. Individual points represent individual animals. Boxes represent the inner quartile, and whiskers represent the minimum and maximum values. **e** Quantification of Pax7-positive cells per millimeter square in TA cross-sections of vehicle or tumor-bearing animals. Individual points represent individual animals. Boxes represent the inner quartile, and whiskers represent the minimum and maximum values. **f** Quantification of centrally located nuclei per myofiber in TA cross-sections. Individual points represent individual animals. Boxes represent the inner quartile, and whiskers represent the minimum and maximum values. **g** Heat map depicting 16 transcripts assessed by qPCR in gastrocnemius muscle of vehicle or tumor-bearing animals. Transcripts are sorted by the highest expression average of tumor sample from top to bottom. Orange = high expression, blue = low expression. *p < 0.05 by Student’s *t* test *n* = 5, 7-week-old male 129S2/SvPasCrl mice for each group

Considering both the loss of lean mass by echoMRI analysis, the accumulation of classical markers of CAW in the serum, and transcriptional changes associated with CAW, we next performed a histological and molecular assessment of skeletal muscle. First, we assessed tibialis anterior (TA) muscle cross sections by immunofluorescent staining for laminin, a marker of myofiber membranes, and DAPI (nuclei) (Fig. 4a). Minimum feret diameters were measured, compiled, and calculated using MyoVision (mean feret diameter: 3 million = 40.4 μm, vehicle = 44.4 μm), and found a statistically significant decrease in the non-linear regressions to fit the histograms of minimum feret diameter distribution of tumor-bearing mice (Fig. 4b) [26]. Additionally, we observed a significant decrease in TA weight in tumor-bearing animals; this was not due to a reduction in the total number of myofibers in the TA of these animals (Fig. 4c, d). This observed decrease in TA weight did not significantly affect grip strength at survival endpoints (Supplemental Figure 3) although tumor-bearing mice were more likely to refuse to grip and were thus excluded from analysis (see the “Methods” section for analysis details/exclusion criteria). Since we observed a reduction in myofiber size, but not the total number of myofibers, we postulated that this could be due to a decrease in the regenerative capacity of muscle. Using immunofluorescent staining, we queried the number of Pax7-positive muscle progenitor cells. We observed a statistically significant increase in Pax7-positive cells per millimeter squared of tissue (Fig. 4e). Additionally, we assessed the number of centrally located nuclei (CLN), which mark actively regenerating myofibers containing recently fused muscle progenitor cells. We did not see any difference in the number of CLN per millimeter square of tissue between vehicle and tumor-bearing mice (Fig. 4f). Lastly, to acquire a molecular understanding of regenerative capacity of muscle from tumor-bearing animals, we used quantitative PCR to measure several transcripts associated with early myogenesis, late myogenesis/fusion, and muscle atrophy. Visualized in a heat map, we see a trend toward higher expression of atrophy transcripts (Trim63 and Fbxo32) and early makers of myogenesis (Pax7 and Myod1), and lower expression of late markers of myogenesis (Fig. 4g).

### Transplanted cell number correlates with survival and CAW progression

**Fig. 5:**
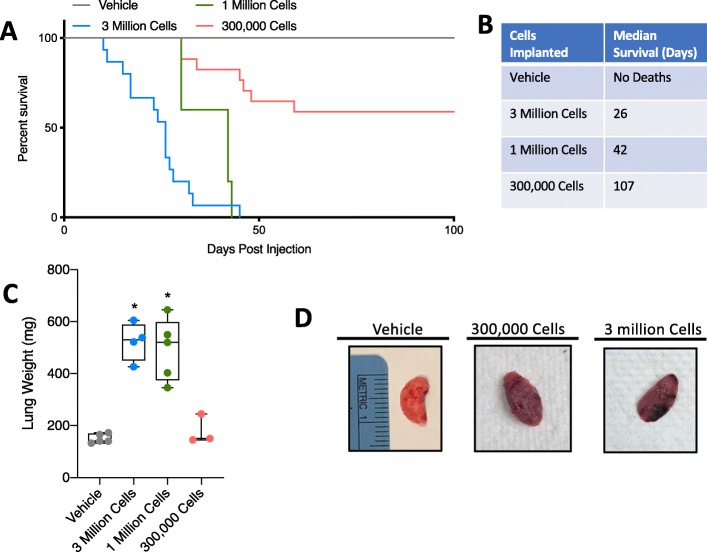
Tumor cell number titration modulates survival time. **a** Survival curve for mice injected with vehicle or a range of tumor cells (300,000, 1 million, or 3 million). **b** Table depicting the median survival for each group. **c** Total lung weight (all lobes) in milligrams for each group. Individual points represent individual animals. Boxes represent the inner quartile, and whiskers represent the minimum and maximum values. *p < 0.05 Student’s *t* test. *n* = 5, vehicle; 4, 3 million; 5, 1 million; 3, 300,000 7-week-old male 129S2/SvPasCrl mice. **d** Representative images of lungs from vehicle, 300,000 cell and 3 million cell groups. Ruler reference is in centimeters

Upon establishing that our lung cancer model induces loss in total body weight and lean mass and is also associated with an impaired muscle regeneration phenotype, we sought to determine if survival time/CAW progression could be experimentally modulated. To accomplish this, we varied the number of cells injected as follows: 3 million cells (used in all previous figures), 1 million cells, 300,000 cells, and vehicle. We also tested 30,000 and 3000 cells but found inconsistent inoculation of tumors and omitted these groups from further study (data not shown). We assessed survival of animals in each group and found that lowering the number of cells injected prolonged survival as evidenced by a statistical increase in survival from 3 million to 1 million cells (*p* = 0.0208), 1 million to 300,000 cells (*p* = 0.0024), and 300,000 cells to vehicle (*p* = 0.0046) (Fig. 5a, b). We also observed changes in lung condition, as evidenced by increased lung weight in 3 and 1 million injected cells, and macroscopic changes in lung condition similar to those observed previously (Fig. 5c, d).

**Fig. 6:**
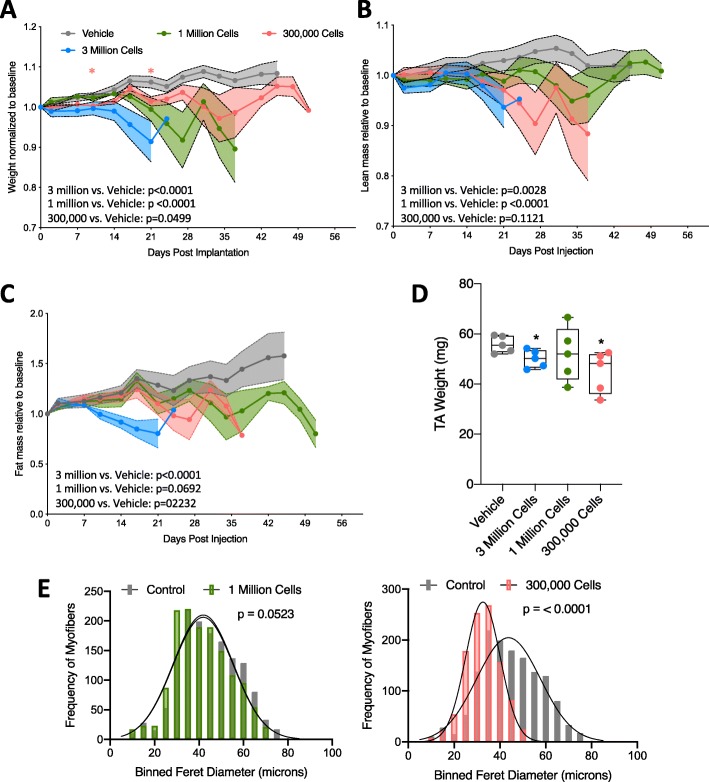
Tumor-bearing mice exhibit progressive tissue loss regardless of initial transplanted cell number. **a** Total mouse weight across study, normalized to pre-tumor baseline weight. Error bars are SEM, and statistical analysis is as described in Fig. 1f. **b** echoMRI calculated total fat mass across study, normalized to pre-tumor baseline fat mass. Error bars are SEM, and statistical analysis is as described in Fig. 1f. **c** echoMRI calculated total lean mass across study, normalized to pre-tumor baseline lean mass. Error bars are SEM, and statistical analysis is as described in Fig. 1f. **d** TA weight in milligrams for each group in study. Individual points represent individual animals. Boxes represent the inner quartile, and whiskers represent the minimum and maximum values. **e** Quantification of minimum feret diameters from muscles of tumor-bearing (1 million cells or 300,000 cells) and vehicle-treated mice. Feret diameters were binned to a histogram and fit with a non-linear regression (Gaussian, least squares regression). Myofibers from tumor-bearing mice were smaller, *p* values calculated by extra sum-of-squares *F* test. One million vs. vehicle (left, *p* = 0.0523), 300,000 vs. vehicle (right, *p* < 0.0001). *n* = 5, vehicle; 4, 3 million; 5, 1 million; 3, 300,000 7-week-old male 129S2/SvPasCrl mice

We next performed body composition and muscle morphometrics analyses in mice receiving variable numbers of tumor cells. As before, we observed decreasing trends in body mass, lean mass, and fat mass in tumor-bearing mice of all groups (Fig. 6a–c). Longitudinal weight, lean, and fat mass data for individual mice are available in Supplemental Figure 4. Specifically, we observed a significant difference in time and treatment co-variance for body weight, in 3 million, 1 million, and 300,000 implanted cell groups compared to vehicle (Fig. 6a). We also observed a significant difference in time and treatment co-variance for total lean mass in 3 million and 1 million implanted cell groups compared to vehicle (Fig. 6b). In lower implantation doses, we did not observe significant differences in total fat mass. Because tumors were restricted primarily to the lungs (with the exception of some metastases) (Table 1), it was not possible to discount tumor mass from echoMRI measurements. Evaluation of TA muscles from tumor-bearing mice revealed a statistically significant decrease in TA mass in the 300,000 cells group, similar to the 3 million cells group (Fig. 6d). Additionally, MyoVision-based assessment of minimum feret diameters revealed increasingly significant decreases as the cell injection number decreased (mean feret diameter: 1 million = 41.3 μm, 300,000 = 32.6 μm, vehicle = 44.4 μm) (Fig. 6e).

**Table 1:** Metastatic characteristics of 393P lung cancer-associated wasting model

Group	% with lung tumor	% with other tumors	% with metastasis in the thoracic cavity
3,000,000 (*n* = 15)	93	33 lower back 10 hind limb	73
1,000,000 (*n* = 5)	100	100	100
300,000 (*n* = 15)	67	33 lower back	53
Vehicle (*n* = 10)	0	0	0

Percentage of animals bearing nodular tumors visible with the naked eye during necropsy and qualitative assessment of necropsied animals showing metastases in the thorax, lower back, and upper hind limb regions

## Discussion

In order to gain a more thorough understanding of CAW, there is a need for models that more accurately reflect the patient condition. This new and novel model for CAW addresses several needs in the field such as: (1) immunocompetence (i.e., a syngeneic model), (2) exhibits both heightened catabolism and suppressed regeneration, (3) exhibits both lean and fat mass loss, (4) exhibits an inflammatory microenvironment, (5) is flexible in timing and severity, and (6) arises in the tumor organ of origin. To the last point, recent studies have highlighted the importance of tumor location on both tumor development and CAW [27–29]. Our model is an easy and effective method to induce tumor formation in the lungs and concomitant CAW, using lung adenocarcinoma cells.

With regard to points two through four, developing therapeutics for CAW is ultimately dependent on mouse models that reflect the complex molecular nature of this syndrome. We showed that this model exhibits an inflammatory signature similar to published signatures of CAW patients [22, 23, 30–32]. Our model has the added attraction that inflammatory status is not affected by tumor implantation, as our model does not require a major surgery or viral infection, known modulators of inflammatory status [33]. Additionally, our model supports numerous studies linking both elevated catabolism and impaired skeletal muscle regeneration as contributors to CAW [5–7, 34]. Our observation that there is an accumulation of Pax7+ cells in the TA muscle, but no change in the number of centrally located nuclei, may be indicative of increased progenitor cell expansion, with failure to incorporate into the damaged/cachectic muscle. Although we probed genes related to fusion and progenitor cell maturation by qPCR and did not observe differences in expression, a more detailed assessment of the progenitor cell population would be necessary to make definitive conclusions about suppressed muscle regeneration in this model.

A major weakness of many CAW mouse models is the age of wasting onset. Genetic models, like the KPC model, are predisposed to spontaneously develop tumors, which gives investigators little control over the age of onset [13]. Here, disrupted muscle development may be a contributor to the loss of skeletal muscle mass in spontaneous genetic models, highlighting the difference between true atrophy and absence of growth. This principle also applies to many transplantation-based models of CAW [35]. Although muscle development is typically considered complete at 3 months, this developmental stage/age is unrepresentative of the patient population [13, 36, 37]. With cachectic cancers affecting a primarily elderly population, it is critical that new models are able to address this confounding factor. For pancreatic cancer, the KPP mouse has addressed this problem by expressing pancreas-specific oncogenes under Cre recombinase control [13]. However, for lung cancer, genetic models of CAW are only beginning to be explored [15]. Although our model has not yet been adapted for use in aged mice, it is amenable to assessing CAW in any age, including elderly (> 18 months old) mice. This may lead to a better understanding of CAW mechanisms and more efficient translation to the clinic.

Another aspect of the patient condition poorly modeled in rodents is the severity and duration of wasting symptoms. Recently, it was established that cachexia syndrome could be divided into three clinical stages: pre-cachexia, cachexia, and refractory cachexia [38]. Although refractory cachexia is marked by a short (only 3 months) survival, wasting is present for much longer—an important observation that is difficult to study in rapidly progressing rodent CAW models. Importantly, with respect to intervention, some suggest that it is in the earlier stages (non-refractory) that therapeutics may be more effective [39]. Despite these features in patients, mouse models like LLC and C26 have rapid symptom onset and short survival times. Failing to capture the earlier stages of cachexia is likely a contributor to the failed clinical trials based on pre-clinical data derived using these models. The ability of our model to be titrated by cell dosage provides an important opportunity to explore the timing and duration of wasting symptoms more thoroughly.

## Conclusions

Despite the many strengths highlighted in this study, the most notable limitation is one common to all orthotopic lung cancer models, which is the difficulty for assessing tumor burden in living animals. This could be remediated by stably transducing cells with a fluorescent/bioluminescent reporter and utilizing in vivo imaging modalities. Additionally, in some cohorts, we observed high levels of metastasis outside the thoracic cavity. This may be representative of patients that exhibit metastases, but also complicates data interpretation. Nevertheless, the model presented here is an important step forward as CAW research progresses and will be a valuable resource for future research aimed at understanding the etiology of lung cancer-associated muscle and fat wasting.

## Supplementary information

**Supplemental Figure 1:** Assessment of Cardiac fibrosis. **(A)** Comparison of percent fibrotic areas in whole heart longitudinal sections from vehicle and tumor-bearing mice. There was no significant difference in percent fibrotic area. **(B)** Representative images of a heart longitudinal section (left), and zoomed in area of fibrosis (right) from a tumor-bearing mouse. **(C)** Representative images of a heart longitudinal section (left), and zoomed in area of fibrosis (right) from a vehicle mouse. Black arrows are marking areas of positive staining for fibrosis.

**Supplemental Figure 2:** Explained variance for the principle component analysis (PCA). PCA analysis and explained variance were generated in TIBCO Spotfire.

**Supplemental Figure 3:** Grip strength at survival endpoint. Grip strength was tested for tumor-bearing and vehicle mice at the survival endpoint for the tumor-bearing mice (vehicle mice were also sacrificed at this point). Each mouse had 3 grips that were averaged into one data point plotted above. Each data point plotted corresponds to one mouse. No statistical significance was found between groups. N=3 vehicle, 4 tumor-bearing.

**Supplemental Figure 4:** Longitudinal body composition assessment for individual mice. Total mouse weight across study, normalized to pre-tumor baseline weight. Each line represents an individual animal in the following groups: Vehicle **(A)**, 3 million injected cells **(D)**, 1 million injected cells **(G)**, 300,000 injected cells **(J)**. echoMRI calculated total fat mass across study, normalized to pre-tumor baseline fat mass. Each line represents an individual animal in the following groups: Vehicle **(B)**, 3 million injected cells **(E)**, 1 million injected cells **(H)**, 300,000 injected cells **(K)**. echoMRI calculated total lean mass across study, normalized to pre-tumor baseline lean mass. Each line represents an individual animal in the following groups: Vehicle **(C)**, 3 million injected cells **(F)**, 1 million injected cells **(I)**, 300,000 injected cells **(L)**. n= 5 vehicle, 3 million injected cells, and 1 million injected cells; 300,000 injected cells. 7-week-old male 129S2/SvPasCrl mice for all groups.

## Data Availability

The datasets used during the current study are available from the corresponding author on a reasonable request.

## Abbreviations

CAW: Cancer-associated muscle wasting
CLN: Centrally located nuclei
DAPI: 4′,6-Diamidino-2-phenylindole
echoMRI: Echo magnetic resonance imaging
Fbxo32: F-box only protein 32
GEMMs: Genetically engineered mouse models
GR: Gastrocnemius
H&E: Hematoxylin and eosin
IACUC: Institutional Animal Care and Use Committee
IL6: Interleukin 6
LIX: C-X-C motif ligand 5
LLC: Lewis lung carcinoma
MyoD1: Myogenic differentiation 1
NCI: National Cancer Institute
NIAMS: National Institute of Arthritis and Musculoskeletal and Skin Diseases
NIH: National Institutes of Health
Pax7: Paired box 7
PCA: Principal component analysis
qPCR: Quantitative polymerase chain reaction
RNAseq: Ribonucleic acid sequencing
RSTP: Regenerative Sciences Training Program
SD: Standard deviation
SEM: Standard error of the mean
TA: Tibialis anterior
TNFα: Tumor necrosis factor alpha
Trim63: Muscle RING finger 1 (MuRF1)
VEGF: Vascular endothelial growth factor
