# Supplementary figures and images for: A novel transplantable model of lung cancer-associated tissue loss and disrupted muscle regeneration
Source: Skelet Muscle. 2020 Mar 9;10:6. doi: 10.1186/s13395-020-00225-6 (PMC7063717; doi:10.1186/s13395-020-00225-6)

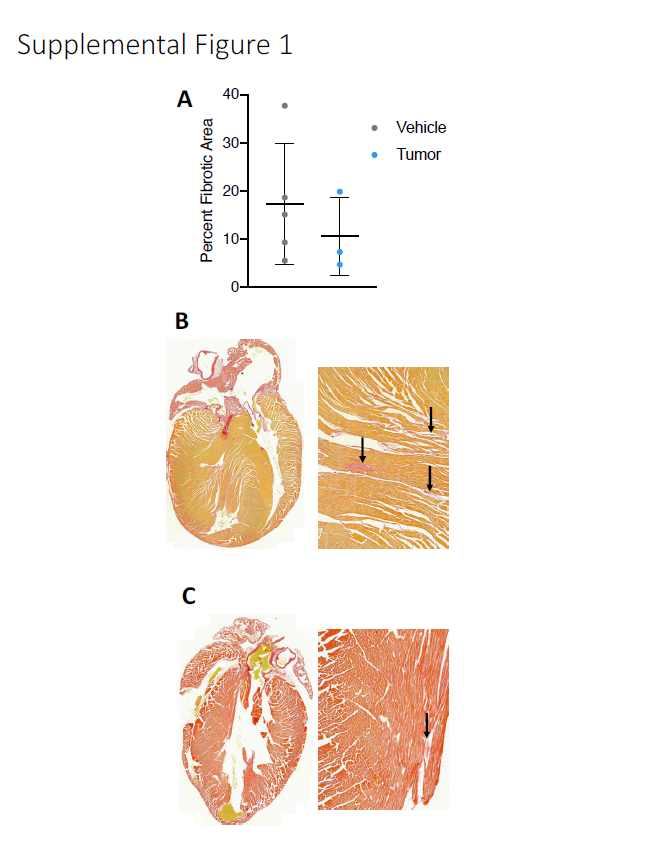

Supplement: Supplementary file 1 — Supplemental Figure 1: Assessment of Cardiac fibrosis. (A) Comparison of percent fibrotic areas in whole heart longitudinal sections from vehicle and tumor-bearing mice. There was no significant difference in percent fibrotic area. (B) Representative images of a heart longitudinal section (left), and zoomed in area of fibrosis (right) from a tumor-bearing mouse. (C) Representative images of a heart longitudinal section (left), and zoomed in area of fibrosis (right) from a vehicle mouse. Black arrows are marking areas of positive staining for fibrosis. [file 13395_2020_225_MOESM1_ESM.docx]

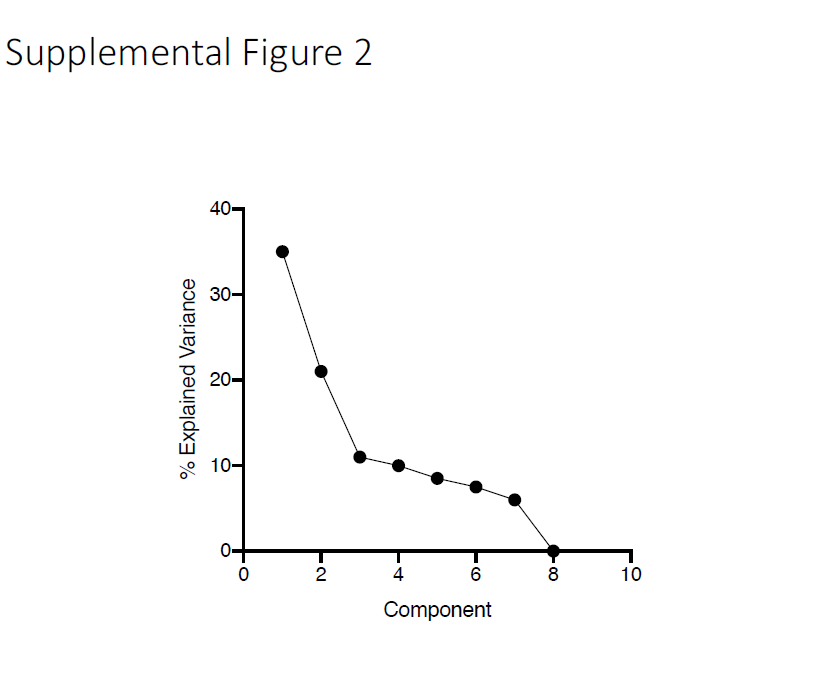

Supplement: Supplementary file 2 — Supplemental Figure 2: Explained variance for the principle component analysis (PCA). PCA analysis and explained variance were generated in TIBCO Spotfire. [file 13395_2020_225_MOESM2_ESM.docx]

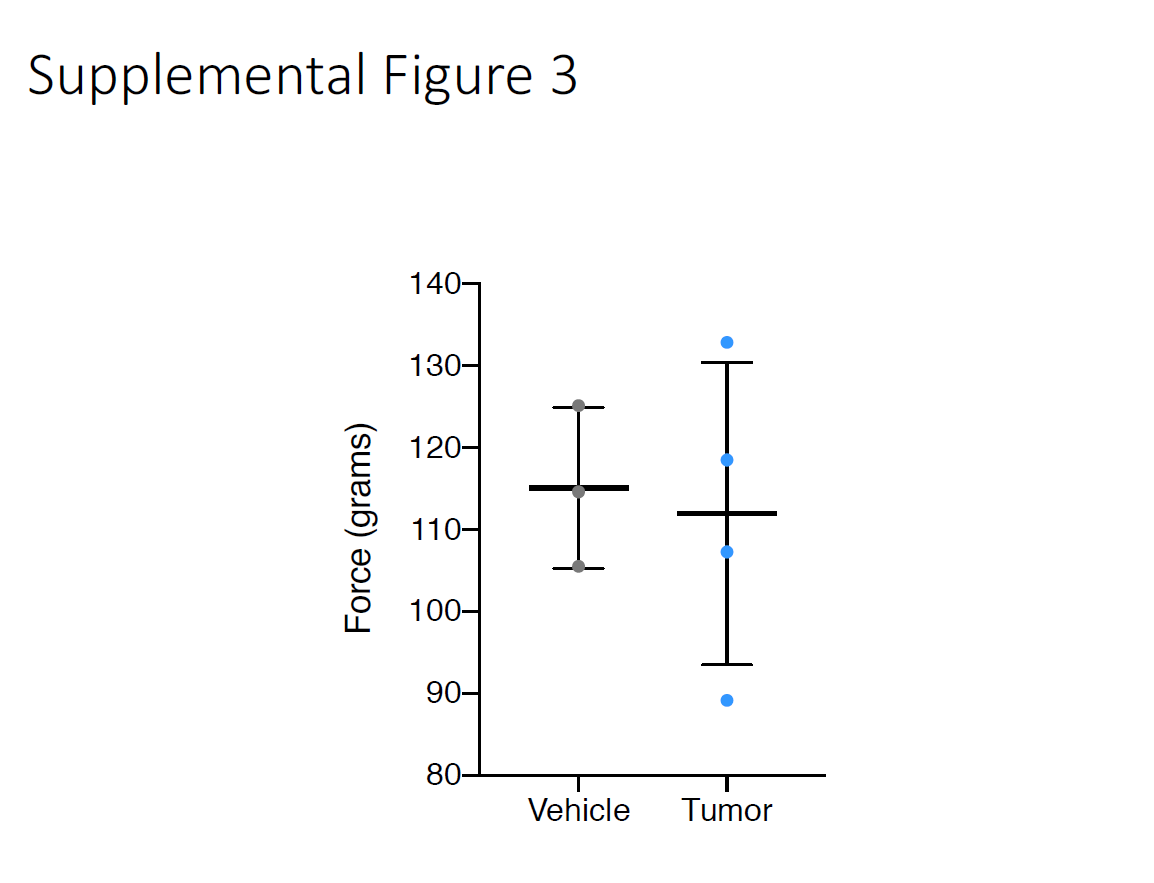

Supplement: Supplementary file 3 — Supplemental Figure 3: Grip strength at survival endpoint. Grip strength was tested for tumor-bearing and vehicle mice at the survival endpoint for the tumor-bearing mice (vehicle mice were also sacrificed at this point). Each mouse had 3 grips that were averaged into one data point plotted above. Each data point plotted corresponds to one mouse. No statistical significance was found between groups. N=3 vehicle, 4 tumor-bearing. [file 13395_2020_225_MOESM3_ESM.docx]

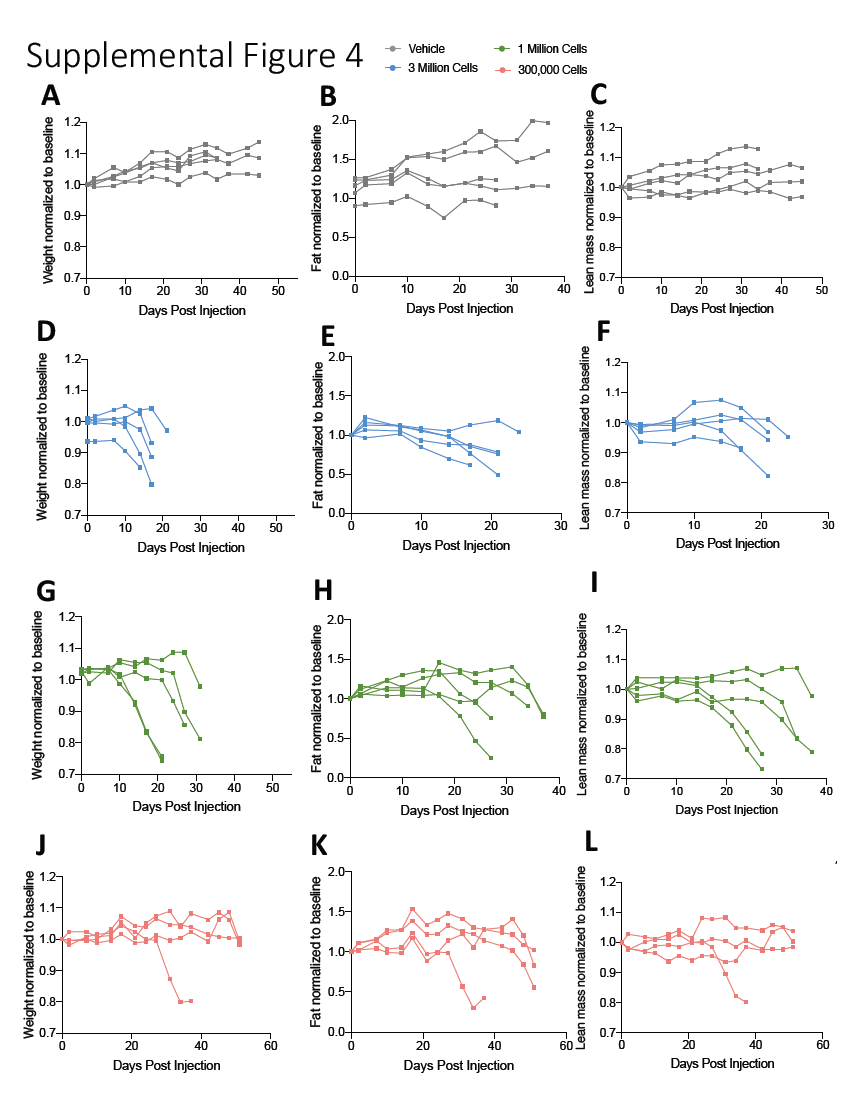

Supplement: Supplementary file 4 — Supplemental Figure 4: Longitudinal body composition assessment for individual mice. Total mouse weight across study, normalized to pre-tumor baseline weight. Each line represents an individual animal in the following groups: Vehicle (A), 3 million injected cells (D), 1 million injected cells (G), 300,000 injected cells (J). echoMRI calculated total fat mass across study, normalized to pre-tumor baseline fat mass. Each line represents an individual animal in the following groups: Vehicle (B), 3 million injected cells (E), 1 million injected cells (H), 300,000 injected cells (K). echoMRI calculated total lean mass across study, normalized to pre-tumor baseline lean mass. Each line represents an individual animal in the following groups: Vehicle (C), 3 million injected cells (F), 1 million injected cells (I), 300,000 injected cells (L). n= 5 vehicle, 3 million injected cells, and 1 million injected cells; 300,000 injected cells. 7-week-old male 129S2/SvPasCrl mice for all groups. [file 13395_2020_225_MOESM4_ESM.docx]
